# Mast cell promotes obesity by activating microglia in hypothalamus

**DOI:** 10.3389/fendo.2025.1544213

**Published:** 2025-03-21

**Authors:** Wen Tian, Jinghui Wang, Yangyang Zhu, Yi Zhang, Liwei Chen, Cheng Hu

**Affiliations:** ^1^ Jinzhou Medical University Graduate Training Base (Shanghai Sixth People’s Hospital Affiliated to Shanghai Jiao Tong University School of Medicine), Jinzhou, China; ^2^ Department of Endocrinology, Xihua Xian People’s Hospital, Zhoukou, China; ^3^ Shanghai Diabetes Institute, Shanghai Key Laboratory of Diabetes Mellitus, Shanghai Clinical Centre for Diabetes, Clinical Research Center, Shanghai Sixth People’s Hospital Affiliated to Shanghai Jiao Tong University School of Medicine, Shanghai, China

**Keywords:** obesity, diabetes, mast cells, microglia, hypothalamus, POMC neuron

## Abstract

**Background:**

Obesity has become a significant public health issue, yet its underlying mechanisms remain complex. The hypothalamus, a crucial part of the central nervous system, plays a vital role in maintaining energy balance. Disruptions in hypothalamic homeostasis can lead to obesity and related metabolic disorders. Recent studies have increasingly focused on the role of intercellular interactions within the hypothalamus in obesity development, though the exact mechanisms are still under investigation. Mast cells, as innate immune cells, have been linked to obesity, but their specific roles and mechanisms require further exploration. This study aims to investigate whether hypothalamic mast cells influence microglia and subsequently affect metabolic homeostasis.

**Methods:**

We conducted experiments to examine the effects of high-fat diets on mast cells in the arcuate nucleus of the hypothalamus. We analyzed the activation of microglia and the activity of POMC neurons in response to mast cell activation. The study involved feeding mice a high-fat diet and then assessing changes in mast cell populations, microglial activation, and neuronal activity in the hypothalamus.

**Results:**

Our findings indicate that high-fat feeding increases the number of mast cells in the arcuate nucleus of the hypothalamus. These mast cells activate microglia, which in turn suppress the activity of POMC neurons. This suppression promotes appetite and reduces energy expenditure, leading to obesity. The results suggest a direct role of hypothalamic mast cells in the regulation of energy balance and obesity development.

**Discussion:**

This study highlights the regulatory role of mast cells in the hypothalamus in the formation of obesity. By activating microglia and influencing POMC neuron activity, mast cells contribute to metabolic dysregulation. These findings provide a new target for the treatment of obesity and related metabolic diseases, emphasizing the importance of hypothalamic immune interactions in metabolic health. Further research is needed to explore the potential therapeutic applications of targeting mast cells in obesity management.

## 1 Introduction

Obesity prevention is a global public health priority due to the increasing global prevalence of obesity and its associated chronic diseases (1). The root cause of obesity is a long-term imbalance in energy metabolism between excessive calorie intake and underconsumption (2). However, innovative research on the specific metabolically relevant molecular mechanisms that drive the obesity process and the innovative treatment methods are still insufficient.

Mast cells are key components of the immune system, originating from hematopoietic stem cells in the bone marrow and are widely distributed across various tissues, including the skin, respiratory system, and gastrointestinal tract. These cells are known for releasing a variety of bioactive molecules, such as histamine, heparin, cytokines, and chemokines, which play crucial roles in immune responses, inflammatory reactions, allergic responses, and tissue repair (3). Recent studies have highlighted the role of mast cells in various metabolic diseases, particularly obesity, where they contribute to the chronic low-grade inflammation characteristic of this condition (4, 5). In adipose tissue, the number of mast cells is significantly increased in obese individuals and animals, exacerbating the inflammatory environment and promoting the development of insulin resistance and metabolic dysfunction (6). Mast cells not only affect obesity through peripheral immune functions but also contribute to energy balance regulation via their role in the central nervous system (CNS) (7, 8). In the brain, mast cells interact with microglia to modulate neuroinflammation and energy metabolism, influencing the pathways that regulate appetite and body weight (9, 10). The emerging connection between mast cells, obesity, and CNS function provides valuable insights into potential obesity treatments and highlights the complex interplay between immune responses and metabolism (11, 12).

The central nervous system (CNS) plays a significant role in energy metabolism and the development of obesity (13). The hypothalamus, a key area in regulating appetite and energy balance, integrates metabolic signals from the periphery to control body weight and energy expenditure (14). Recent studies have shown that microglia play an essential role in the onset and progression of obesity (15). Microglia are resident immune cells in the CNS that perform immune surveillance in the brain, influencing energy balance and metabolism through the regulation of neuroinflammation (16). Obesity is closely linked to neuroinflammation, with strong neuroinflammatory responses potentially leading to disruptions in appetite regulation and weight gain (17, 18). Microglia secrete pro-inflammatory factors that promote obesity-related neuroinflammation and impact energy metabolism signals in the hypothalamus (19). Moreover, research has increasingly focused on the role of mast cells in the CNS. Mast cells may play a critical role in the onset and progression of obesity by activating microglia and modulating neuroinflammation (20). The finding suggests that mast cells may influence energy regulation in the central nervous system by altering microglial activity (8), offering new directions for obesity treatment research.

In this study, we focused on the central influence of mast cells on energy metabolism. Through this study, we aim to partially explain the specific mechanisms of mast cells in metabolic homeostasis and provide new insights into obesity treatment.

## 2 Result

### 2.1 The number of mast cells and microglia increases in the ARC of high-fat diet-fed mice

**Figure 1:**
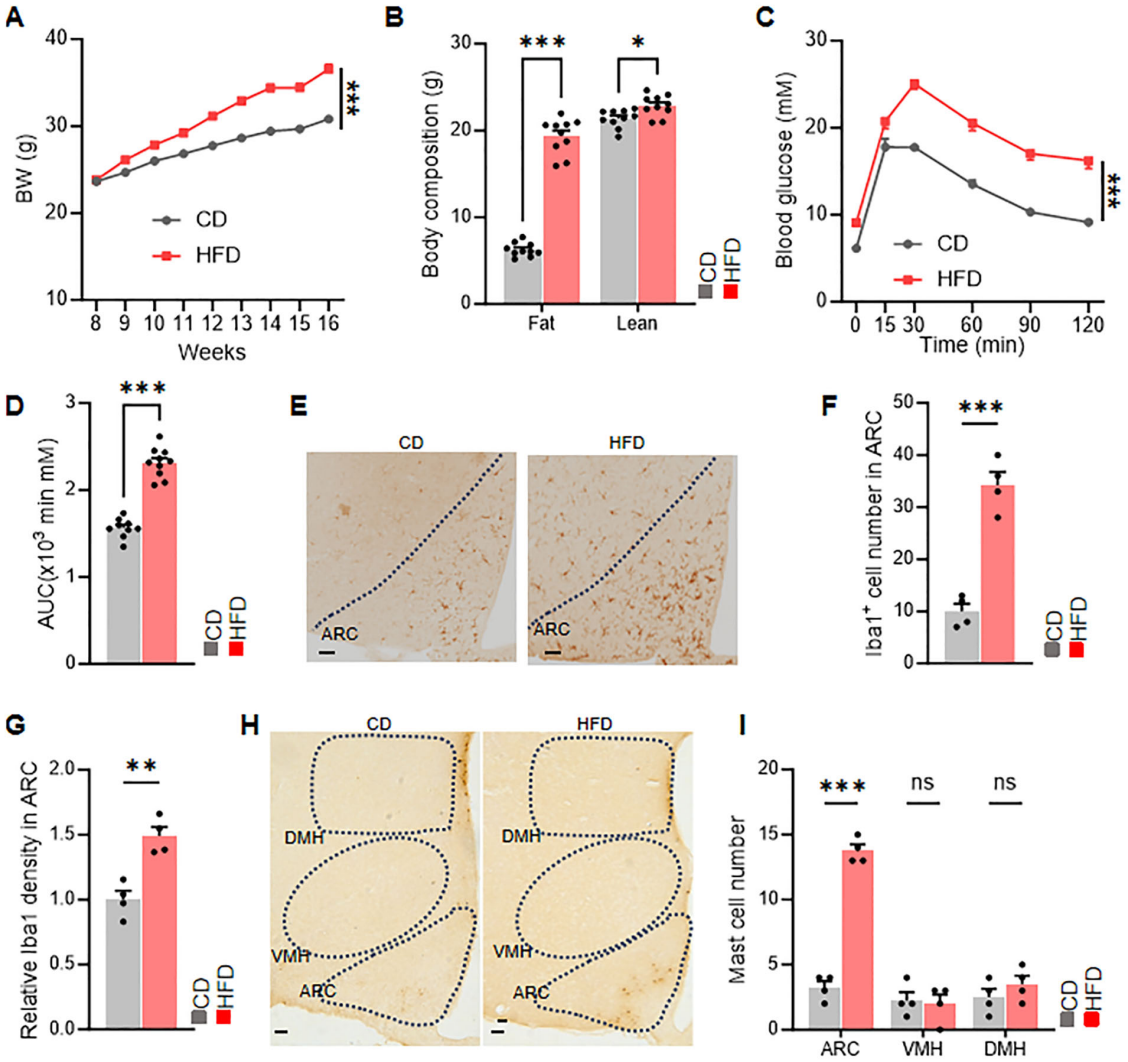
HFD increases the expression of mast cells in the ARC of hypothalamus. **(A)** Body weight changes in chow-fed and HFD-fed mice, n = 10 per group. **(B)** Body composition of chow-fed and HFD-fed mice, n = 10 per group. **(C, D)** Glucose tolerance test (GTT) and area under the curve (AUC), n = 10 per group. **(E-G)** Representative immunohistochemical staining and relative density of microglia in the ARC. **(H, I)** Representative immunohistochemical staining and quantification of mast cells in the hypothalamic ARC, VMH, and DMH nuclei of chow-fed and HFD-fed mice. ns, not significant. Data are presented as mean ± SEM. *p < 0.05, **p < 0.01, ***p < 0.001, two-tailed Student’s t-test **(B, D, F G, I)**, two-way ANOVA with Bonferroni’s *post hoc* test **(A, C)**.

Since obesity is an inflammatory condition, we sought to investigate the relationship between obesity and mast cells by subjecting mice to either a normal chow diet (CD) or a high-fat diet (HFD). Compared with mice fed with CD, mice fed with HFD showed an increase in body weight, with corresponding increases in both fat mass and lean mass (**Figures 1A, B**). Additionally, glucose tolerance tests revealed impaired glucose tolerance in mice fed with HFD (**Figures 1C, D**).

Furthermore, immunohistochemical staining demonstrated an increase in microglia and mast cells in the arcuate nucleus (ARC) of mice fed with HFD (**Figures 1E–I**). These findings underscore the association between mast cells in the ARC and obesity. These findings indicated that mast cell in ARC may relate with metabolic dysfunction.

### 2.2 Genetic deficiency of mast cells improves metabolic homeostasis in HFD mice

**Figure 2:**
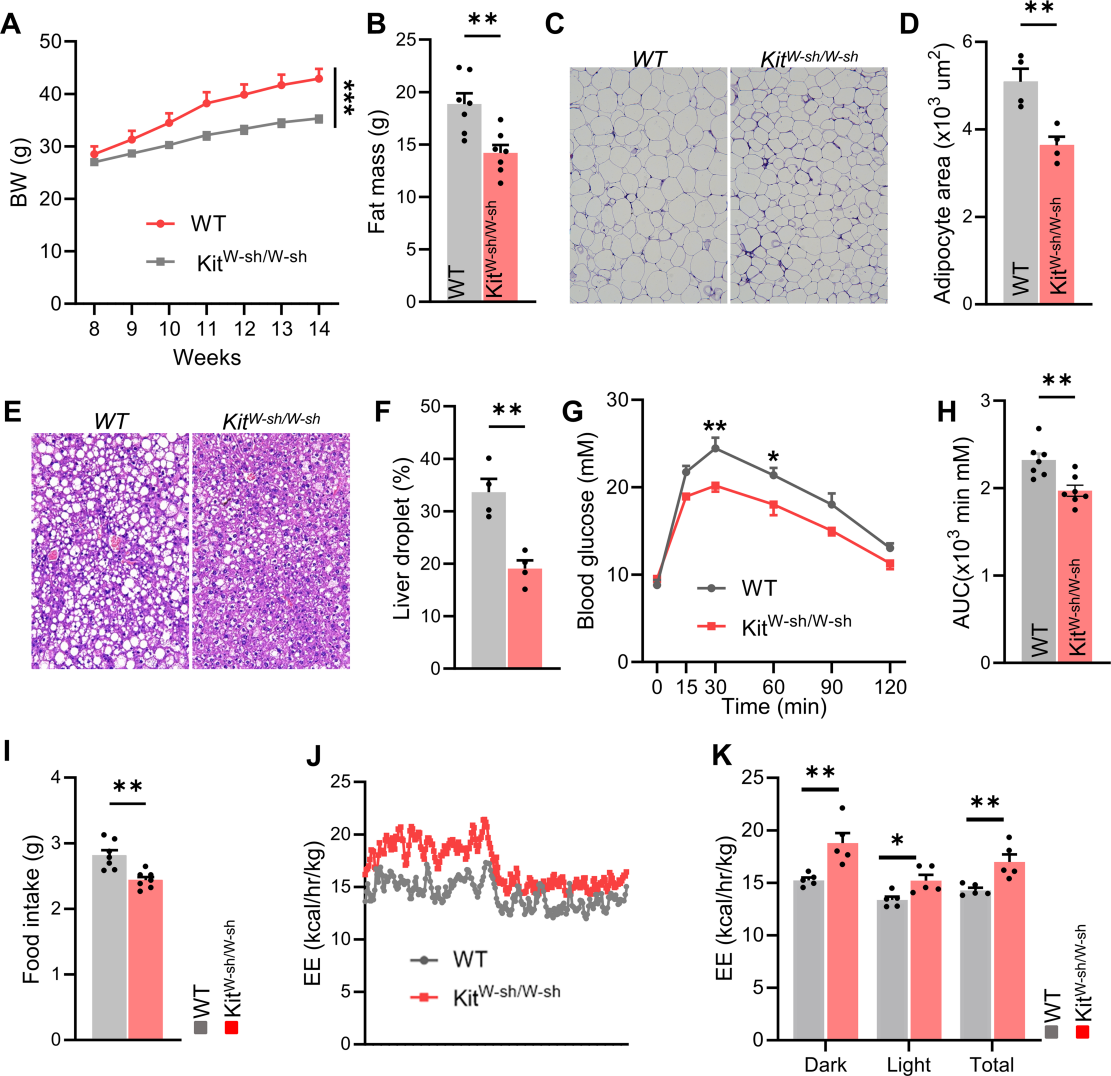
Genetic deficiency of mast cells improves metabolic homeostasis in HFD-fed mice. **(A)** Body weight changes and **(B)** fat mass in control and Kit^W-sh/W-sh mice, n = 7 per group. **(C)** Representative H&E staining images of adipose tissue in control and Kit^W-sh/W-sh mice, and **(D)** average adipocyte size, n = 4 per group. **(E, F)** Representative liver H&E staining and relative lipid droplet area, n = 4 mice per group. **(G, H)** Glucose tolerance test (GTT) and area under the curve (AUC), n = 7 per group. **(I, J)** Food intake and total energy expenditure for both groups, n = 7 per group. Data are presented as mean ± SEM. *p < 0.05, **p < 0.01, ***p < 0.001, two-tailed Student’s t-test **(B, D, F, H, I)**, two-way ANOVA with Bonferroni’s *post hoc* test **(A, G, K)**.

We generated a mouse model Kit^W-sh/W-sh mice with a genetic deficiency in mast cells and then fed with HFD diet. Compared with control mice, mast cell-deficient mice exhibited lower body weight and reduced fat mass (**Figures 2A, B**). The H&E staining results revealed that, compared to the control mice, the adipose tissue of mast cell-deficient mice exhibited a significant reduction in adipocyte size, and the relative lipid droplet content in the liver tissue was also decreased (**Figures 2C–F**). As obesity progresses, glucose homeostasis is typically impaired. Therefore, we further investigated whether reduced mast cell presence could improve glucose metabolism associated with obesity. In glucose tolerance tests, mast cell-deficient mice showed better performance compared with controls (**Figures 2G, H**). Additionally, food intake and metabolic cage experiments indicated reduced food consumption and increased energy expenditure in mast cell-deficient mice (**Figures 2I–K**). These findings suggest that a mast cell deficiency improves metabolic homeostasis under mice fed with HFD.

### 2.3 Pharmacological stabilization of mast cells improves metabolic phenotype in mice

**Figure 3:**
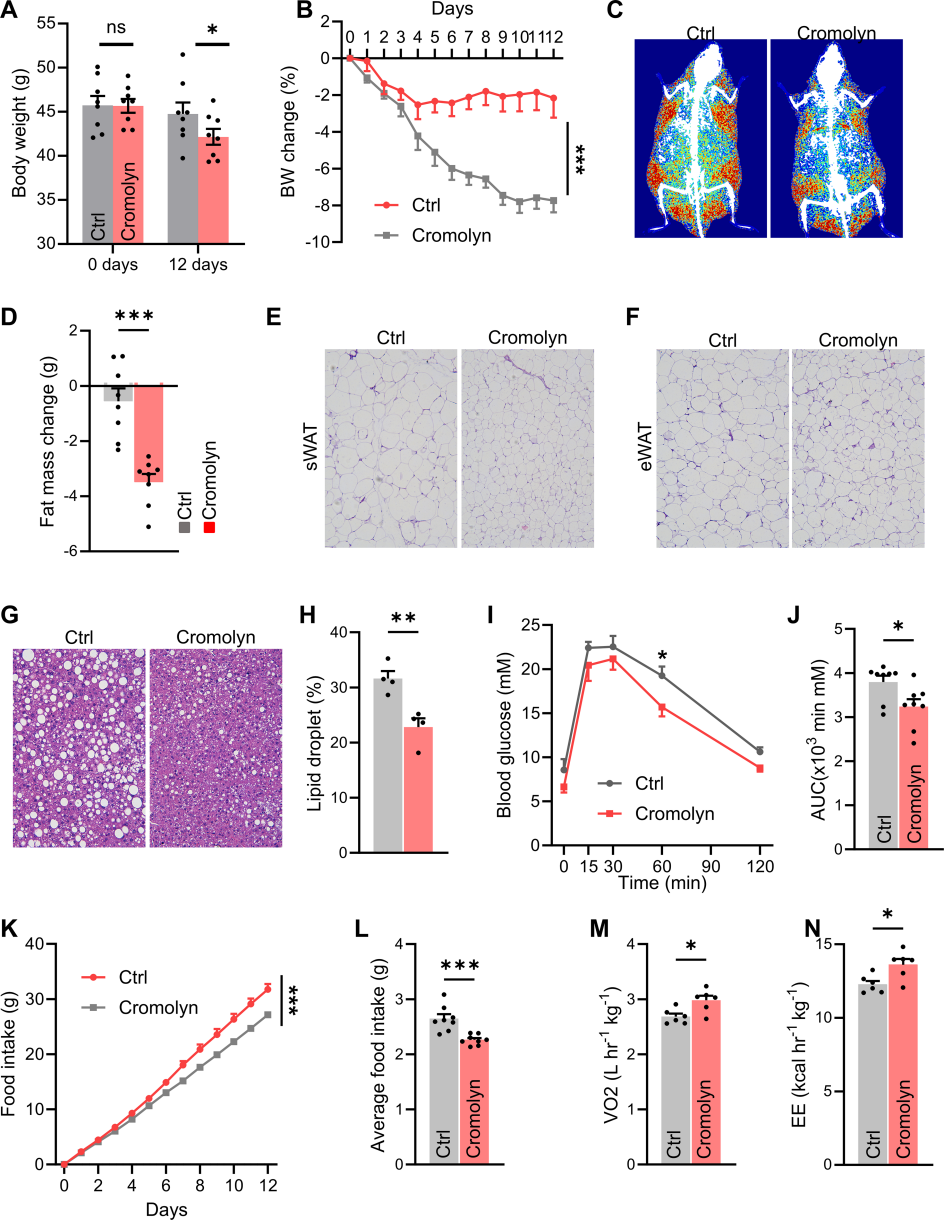
Pharmacological stabilization of mast cells in brain improves metabolic homeostasis in HFD-fed mice. **(A)** Body weight and body weight changes **(B)** in control and cromolyn sodium-injected mice, n = 8 per group. DEXA images **(C)** and fat mass **(D)** of control and cromolyn sodium-injected mice. Representative H&E-stained images of adipose tissue **(E)** and average adipocyte size **(F)**, n = 8 per group. **(G, H)** Representative H&E-stained liver images and relative lipid droplet area, n = 8 per group. **(I, J)** Glucose tolerance test (GTT) and area under the curve (AUC), n = 8 per group. **(K-N)** Food intake and total energy expenditure in both groups of mice, n = 8 per group. ns, not significant. Data are presented as mean ± SEM. *p < 0.05, **p < 0.01, ***p < 0.001, two-tailed Student’s t-test **(D, H, J, L, M, N)**, two-way ANOVA with Bonferroni’s *post hoc* test **(A, I, K)**.

We hypothesized that mast cells act in part through the hypothalamus. To test this, we performed lateral ventricle cannulation surgery on mice fed a HFD for 12 weeks. We then administered all mice a daily intracerebroventricular (i.c.v.) injection of saline (Ctrl) or cromolyn sodium, a mast cell stabilizer, directly into the lateral ventricle for 12 consecutive days. Compared with control mice, those injected with cromolyn sodium showed reduced body weight and significantly lower fat mass (**Figures 3A–D**). The H&E staining results demonstrated that, compared to the control group, the mice treated with sodium cromolyn exhibited a significant reduction in adipocyte size within the adipose tissue, as well as a decrease in the relative lipid droplet content in the liver tissue (**Figures 3E–H**). Additionally, in glucose tolerance tests, cromolyn sodium-treated mice demonstrated better performance compared to controls (**Figures 3I, J**). Furthermore, metabolic cage experiments revealed reduced food intake and increased energy expenditure in cromolyn sodium-treated mice (**Figures 3K, N**). These findings suggest that pharmacological stabilization of mast cells improves metabolic homeostasis in mice fed with HFD.

### 2.4 Microglia mediates the effects of cromolyn sodium on energy balance

Many studies have confirmed that in the central nervous system, mast cells can activate microglial cells, which will subsequently lead to the occurrence of inflammation. Moreover, the activation of microglial cells in the hypothalamus is crucial for the development of obesity. Therefore, we speculated that in the central nervous system, mast cells can cause the impairment of metabolic homeostasis through microglial cells.

**Figure 4:**
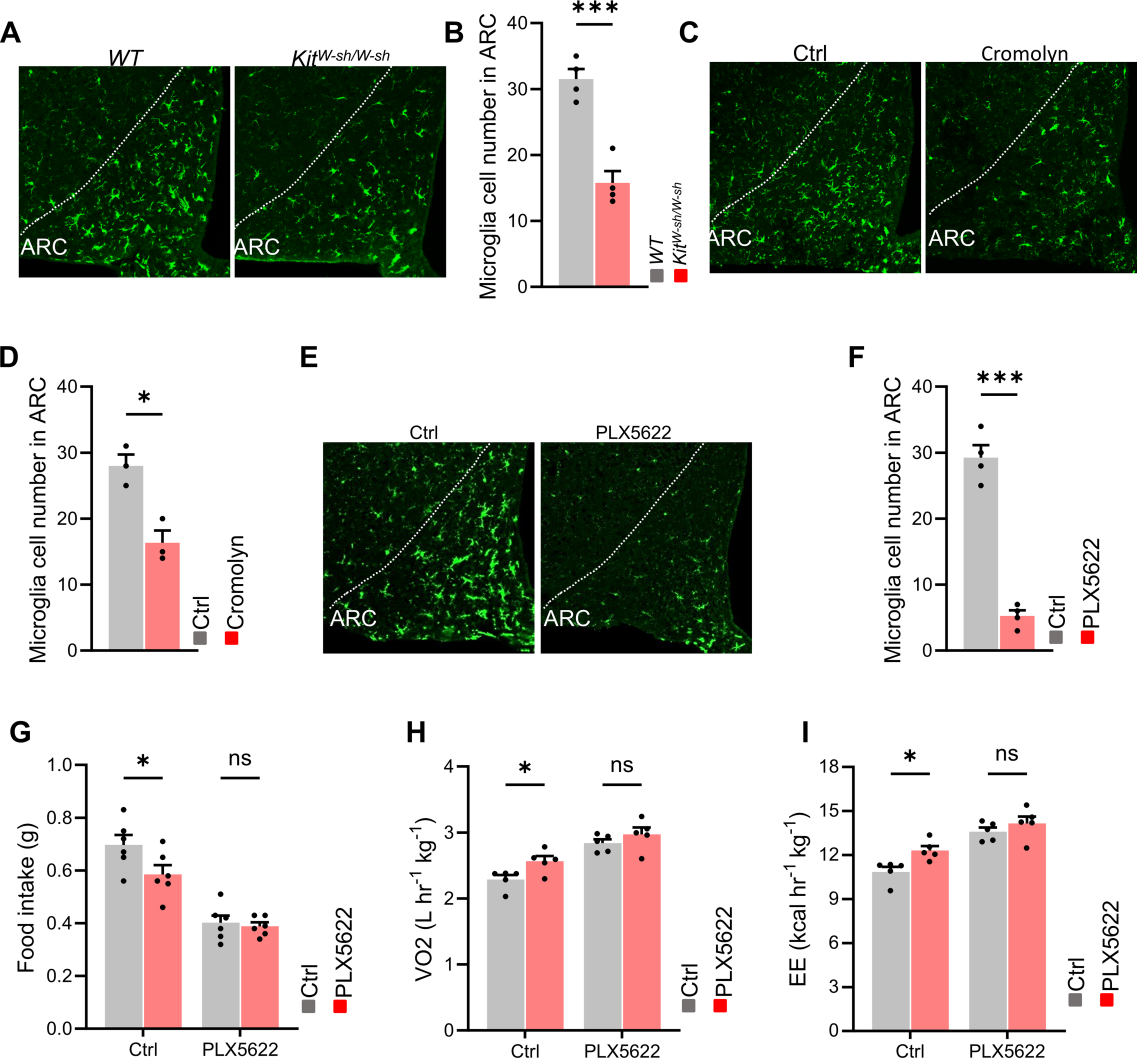
Microglia mediates the effects of cromolyn sodium on energy balance. **(A, B)** Immunostaining and quantification of the microglial marker Iba1 in Kit^W-sh/W-sh mice and control mice. **(C, D)** Immunostaining and quantification of Iba1 in the control and cromolyn sodium-treated mice. **(E, F)** Immunostaining and quantification of Iba1 in control and PLX5622 treated mice. **(G-I)** After intracranial administration of the microglial cell-depleting agent PLX5622, differences in food intake, oxygen consumption, and energy expenditure between the cromolyn-treated group and the control saline group were observed. ns, not significant. Data are presented as mean ± SEM. *p < 0.05, **p < 0.01, ***p < 0.001, two-tailed Student’s t-test **(B, D, F)**, two-way ANOVA with Bonferroni’s *post hoc* test **(G-I)**.

To investigate the impact of mast cells on microglial activation, we used immunostaining to detect Iba1, the microglial marker, expression. In Kit^W-sh/W-sh mice, number of microglia in the ARC of the hypothalamus was significantly reduced compared to control mice (**Figures 4A, B**). A similar reduction in microglial number was observed in mice injected with cromolyn sodium (**Figures 4C, D**). To further confirm that microglia were involved in the role of mast cells on metabolic homeostasis, we used PLX5622.PLX5622 is a highly selective, blood-brain barrier-penetrating, and orally active microglial inhibitor. It can be used both before and during disease progression to achieve extensive and specific depletion of microglia. Immunostaining for Iba1 confirmed a reduction in microglia (**Figures 4E, F**). We found that the role of cromolyn sodium on food intake and oxygen consumption were largely abolished when mice microglia were eliminated (**Figures 4G–I**). These results suggest that mast cells in the brain regulates energy balance through their effects on microglia.

### 2.5 Pharmacological inhibition of mast cells exerts its effects by activating POMC neurons

**Figure 5:**
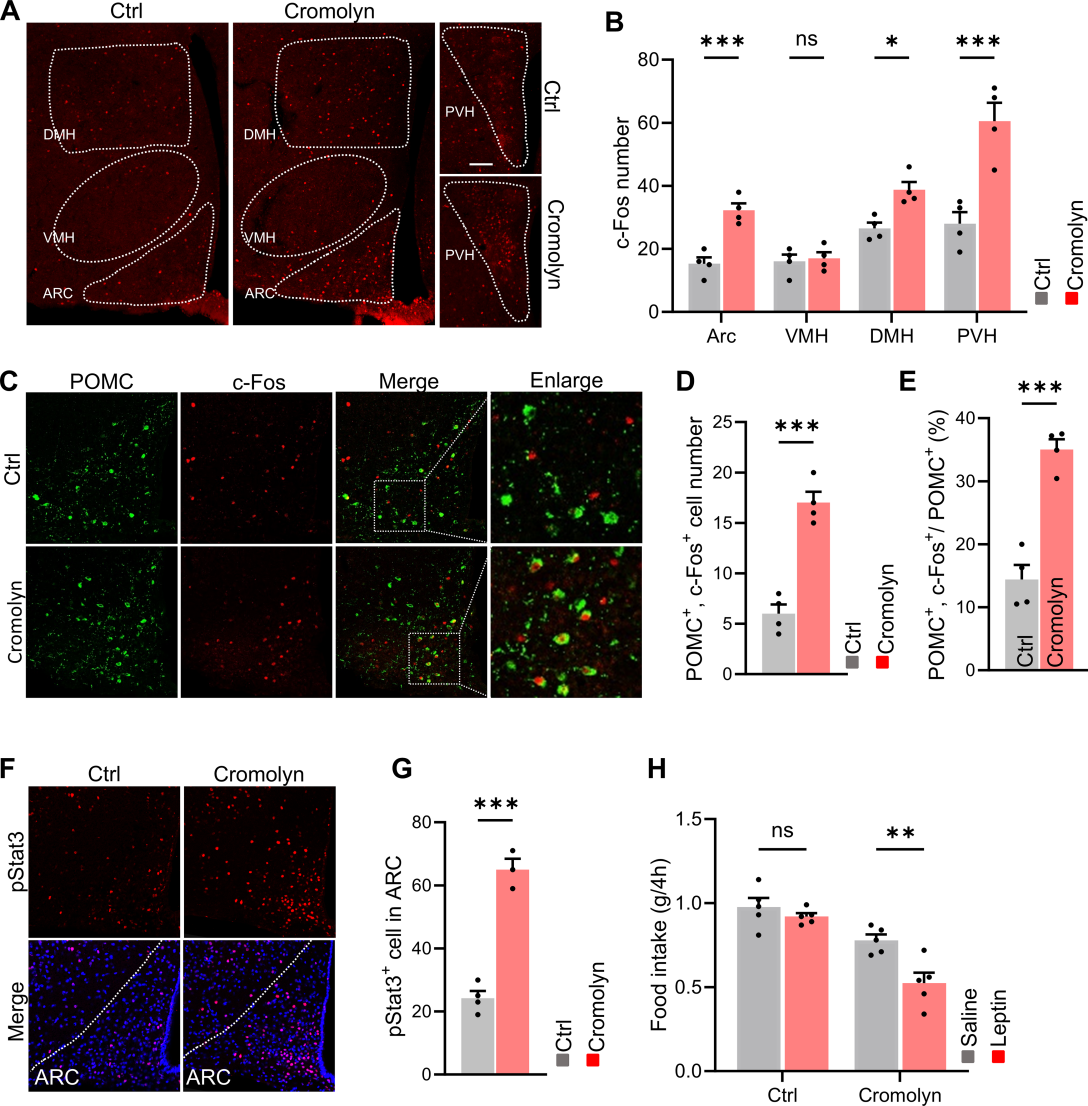
Pharmacological inhibition of mast cells activated POMC neurons and ameliorated leptin resistance. **(A, B)** Immunofluorescence staining of c-Fos in hypothalamus and quantification in control and cromolyn sodium-injected mice. **(C, D)** Immunofluorescence staining for POMC and c-Fos in the ARC brain region and quantification in control and cromolyn sodium-injected mice.**(E, F)** Immunofluorescence staining for p-STAT3 in the ARC brain region and quantification in control and cromolyn sodium-injected mice. **(G)** Food intake after intraperitoneal injection of leptin or saline in control and cromolyn sodium-injected mice. ns, not significant. Data are presented as mean ± SEM. *p < 0.05, ***p < 0.001, two-tailed Student’s t-test **(B, D, E, G)**, two-way ANOVA with Bonferroni’s *post hoc* test **(H)**.

To investigate the downstream neurons affected by mast cells, we performed brain slice staining on cromolyn sodium-injected and control mice. The results showed that, compared to the control group, the number of activated neurons in the arcuate nucleus (ARC), dorsomedial hypothalamus (DMH), and paraventricular nucleus (PVH) of the hypothalamus was significantly increased in the cromolyn sodium-treated group, while no difference was observed in the ventromedial nucleus (VMH) (**Figures 5A, B**). It is well known that the hypothalamus controls various neuroendocrine functions that integrate metabolic feedback and regulate energy homeostasis. A key regulatory function is mediated by the arcuate nucleus melanocortin system, which consists of two functionally antagonistic neuronal populations: POMC and AgRP. Activation of POMC neurons regulates metabolic homeostasis by reducing food intake and increasing energy expenditure through downstream pathways. Next, we performed double immunostaining for POMC and c-Fos in cromolyn sodium-injected and control mice, and found that pharmacological inhibition of mast cells increased the number of activated POMC neurons (**Figures 5C–E**).

### 2.6 Cromolyn sodium ameliorate leptin resistance of HFD mice

Previous studies have shown that leptin can rapidly affect neuronal activity. Under exogenous leptin stimulation, POMC neurons exhibit STAT3 activation, where leptin-stimulated STAT3 dimers bind to the POMC promoter in the nucleus, stimulating POMC expression, which in turn reduces food intake and increases energy expenditure (21).We then performed immunofluorescence staining for p-STAT3, and found that cromolyn sodium-treated mice showed a higher number of STAT3-activated cells, indicating increased sensitivity to leptin (**Figures 5F, G**). More importantly. leptin significantly reduced food intake in cromolyn sodium treated mice fed a HFD, whereas it failed to decrease appetite in control mice (**Figure 5H**).

### 2.7 POMC-melanocortin system participated in the effects of cromolyn sodium on energy balance

**Figure 6:**
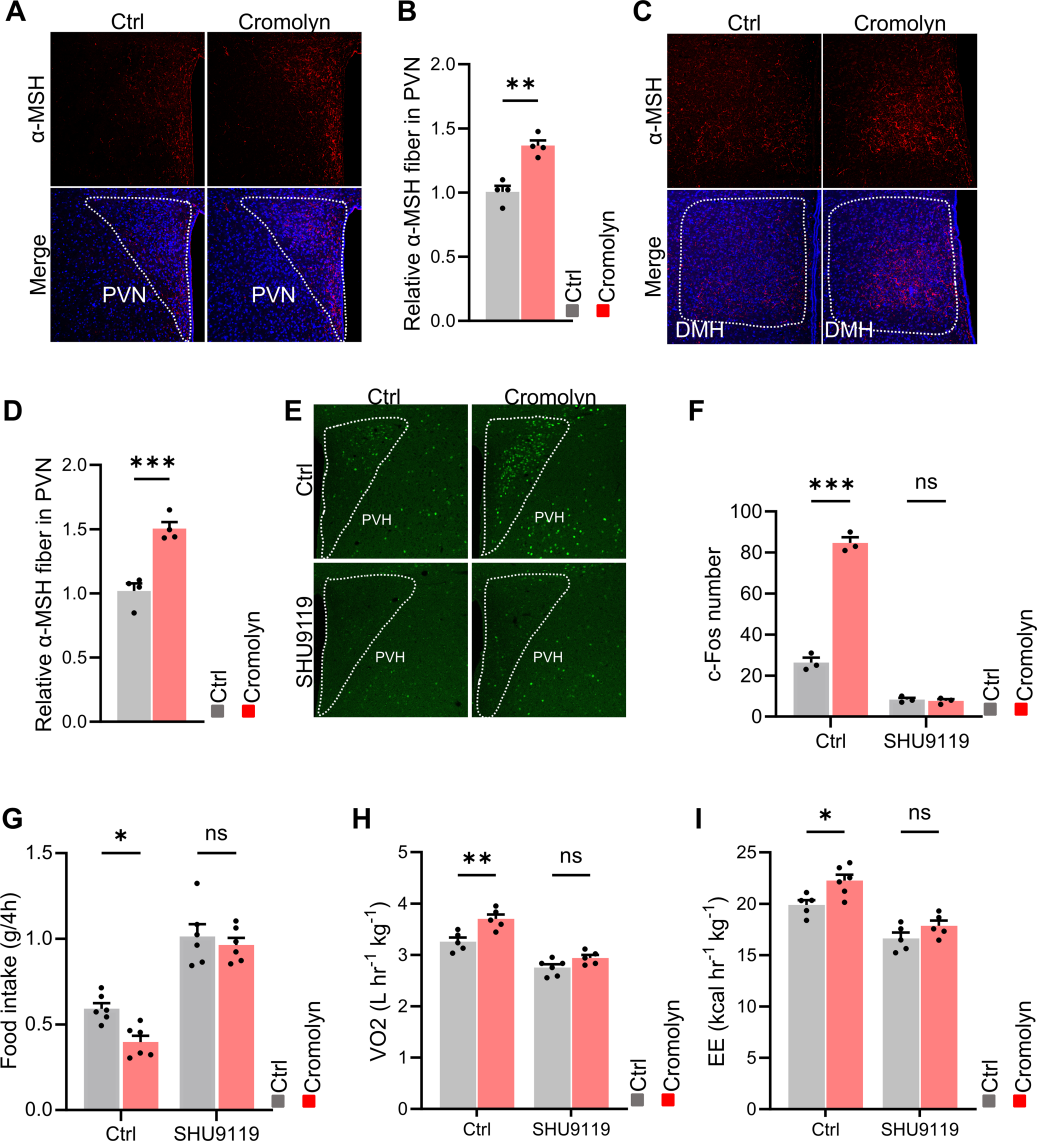
POMC-melanocortin system participated in the effects of cromolyn sodium on energy balance. **(A, B)** Immunofluorescence staining and quantification of α-MSH in the hypothalamic PVN nucleus in brain slices from control and cromolyn sodium-injected mice. **(C, D)** Immunofluorescence co-staining and quantification of α-MSH in the hypothalamic DMH nucleus in brain slices from control and cromolyn sodium-injected mice. **(E, F)** Immunofluorescence staining and quantification of c-Fos in brain slices from control and cromolyn sodium-injected mice, which were i.c.v. saline control and SHU9119. **(G, H)** Food intake, oxygen consumption, and energy expenditure in control and cromolyn sodium-injected mice, as well as saline and SHU9119. ns, not significant. Data are presented as mean ± SEM. *p < 0.05, **p < 0.01, ***p < 0.001, two-tailed Student’s t-test **(B, D)**, two-way ANOVA with Bonferroni’s *post hoc* test **(F-I)**.

Previous studies have shown that POMC neurons in the arcuate nucleus (ARC) release α-MSH at their synaptic terminals, which acts on neurons in the hypothalamus expressing the MC4R receptor. This action helps regulate metabolic homeostasis by reducing food intake and increasing energy expenditure. To investigate this, we performed α-MSH staining on brain slices from control and cromolyn sodium-injected mice. The results showed an increase in α-MSH staining in the hypothalamic PVN and DMH nuclei of cromolyn sodium-treated mice (**Figures 6A–D**).

SHU 9119 is an effective antagonist of the human melanocortin receptors MC3R and MC4R. We found that when SHU 9119 was i.c.v. injected to both cromolyn sodium-injected and control mice, the difference in the number of activated neurons in the hypothalamus disappeared (**Figures 6E, F**). Additionally, the differences in food intake, oxygen consumption, and energy expenditure between the two groups were also eliminated (**Figures 6G–I**). These results suggest that inhibition of mast cell activation can activate POMC neurons, leading to the release of α-MSH at their synaptic terminals. This, in turn, acts on neurons in the hypothalamus expressing MC4R receptors, reducing food intake and increasing energy expenditure to regulate metabolic homeostasis.

## 3 Discussion

Mast cells are tissue-resident effector cells in allergic reactions (22). Like basophils, mast cells express high-affinity IgE binding sites and store numerous pro-inflammatory and vasoactive mediators within their metachromatic granules (23). Unlike common myeloid precursors in the bone marrow, immature mast cell precursors with proliferative potential leave the bone marrow through specific integrins and chemokine receptors, migrating to epithelial tissues in contact with the external environment, such as the skin, respiratory tract, and gastrointestinal tract. They also reside in perivascular spaces around nerves and in connective tissues, where they terminally differentiate into non-proliferative mature cells expressing secretory granules (24). Mast cells are widely distributed across tissues, primarily located at the interfaces between the host and the external environment. They play immunomodulatory and physiological roles within the epithelium, endothelium, and nervous system. Their ubiquitous presence grants mast cells a privileged position, enabling them to act as guardians of the immune system while also participating in numerous biological processes and maintaining homeostasis, such as in tissue repair, angiogenesis, and innate immunity (25). However, when their functions are inadequately regulated, mast cells can interact with the microenvironment and rapidly release a series of mediators, leading to diseases such as allergies, cancer, and obesity (26). Mast cells are one of the two types of resident immune cells in the central nervous system (the other type being microglia), primarily located near the blood-brain barrier and the neurovascular unit (27). Mast cells (MCs) belong to the hematopoietic myeloid lineage and serve as innate immune sentinels for pathogens in peripheral connective tissues and mucosal tissues. Once activated by certain pathogens, MCs release inflammatory mediators, vasoactive molecules, and proteases stored within their granules. Additionally, the recognition of pathogens by MCs triggers the *de novo* production of cytokines, chemokines, and eicosanoids (28).These products of MCs are generally pro-inflammatory and vasoactive, and they can mobilize other innate and adaptive immune cell types to achieve optimal pathogen clearance.

Studies have shown that the white adipose tissue in obese humans contains a large number of mast cells, and serum levels of mast cell tryptase are significantly higher in obese individuals compared to lean subjects, suggesting a role for these inflammatory cells in obesity and diabetes (4). Previous research has mainly focused on peripheral metabolic organs, with less attention to the hypothalamus, which plays a central role in energy balance. In this study, we observed an increase in the number of mast cells in the arcuate nucleus (ARC) of the hypothalamus in obese mice. We also found that genetic deficiency or pharmacological stabilization of central mast cells can improve obesity and related metabolic dysfunctions.

We further explored the specific downstream targets of mast cells’ influence on obesity. Microglia are the resident immune cells in the CNS, typically responding to neuronal injury and removing damaged cells through phagocytosis. Activation of microglia is a hallmark of brain pathology, and inhibiting microglia-mediated inflammation is considered an important strategy for treating neurodegenerative diseases (29). Studies have also shown that inflammatory signaling from microglia determines the immune response of the mediobasal hypothalamus (MBH) to dietary excess and regulates hypothalamic control of energy homeostasis in mice. Pharmacological depletion of microglia or selective inhibition of NF-κB-dependent signaling in microglia significantly limits diet-induced hyperphagia and weight gain (15). In our study, we found that i.c.v. injection of a mast cell stabilizer in mice reduced hypothalamic microglial activation and resulted in weight loss compared to the control group. However, when both groups were subsequently treated with a microglial depletion agent, this difference disappeared. Our findings suggest that mast cell activation influences obesity by activating microglia.

Inactivation of microglia increases the activity of POMC neurons, which subsequently release melanocyte-stimulating hormone (α-MSH) that acts on second-order neurons expressing melanocortin receptors (MC3R/MC4R) to suppress food intake (10, 30) We further validated this mechanism by performing immunofluorescence staining for POMC and α-MSH in mice treated with an intraperitoneal injection of a mast cell stabilizer and in control mice, as well as by observing phenotypes after administering the MC3/4R antagonist SHU 9119. Our results confirm that mast cells influence food intake by activating microglia, which inhibits the release of α-MSH from POMC neurons, thus reducing α-MSH binding to MC4R receptors and attenuating its appetite-suppressing effect. Overall, our findings underscore central mast cells as a potential therapeutic target for obesity and related diseases.

### 3.1 Limitations of the study

There are certain limitations to our study that should be acknowledged. Due to technical constraints, the genetically deficient mice we used do not have hypothalamus-specific deletions. While KitW-sh mice remain the most widely validated mast cell-deficient model, we acknowledge that bone marrow transplantation or Cre-lox systems may provide complementary approaches for specific research contexts. Therefore, we cannot rule out the potential role of mast cells in the peripheral system and in other central neurons or cell types. And it should be noted that the intracerebroventricular (i.c.v.) administration method poses challenges in achieving precise targeting of the hypothalamic region. Sodium cromoglicate may exert effects through actions on areas beyond the hypothalamus, and these factors could potentially influence the experimental outcomes. Additionally, because of technical limitations, we were unable to determine whether the hypothalamic mast cells are inherently present in the CNS or are recruited from the periphery. Lastly, we used tryptase staining for mast cell identification without further confirmation through other methods.

## 4 Experimental section

### 4.1 Ethical approval

Animals were housed under specific pathogen-free (SPF) conditions, with a 12-hour light-dark cycle, and had ad libitum access to standard chow and water. All animal experimental procedures were approved by the Shanghai Sixth Laboratory Animal Management and Use Committee (No:DWLL2024-1055).

### 4.2 The mice

Male C57 BL/6 were purchased from GemPharmatech (Nanjing, China). The KitW-sh/W-sh mice were Kindly shared by Dr. Xin Wang from Shanghai Sixth hospital. Mice housed in a temperature-controlled room (22–24°C) on a 12-hours light/12-hours dark cycle with ad libitum access to water and food unless otherwise stated. Animal experiments were approved by the Institutional Animal Care and Use Committee of Shanghai Sixth People’s Hospital. Regular chow (9.4% kcal from fat) and HFD (60% kcal from fat) were purchased from Xietong Bioscience (Nanjing, China) and Research Diets (New Brunswick, NJ, USA), respectively. The DIO mouse models were fed a HFD for 12 weeks, starting at 6 weeks, unless otherwise noted.

### 4.3 Surgery

Stereotaxic surgery was performed for lateral ventricle cannulation as previously described. Briefly, the mice were anesthetized with avertin (300 mg/kg, Sigma) and placed on a stereotaxic instrument (RWD, Shenzhen, China). Subsequently, a 28 G guide cannula was implanted into the lateral ventricle, 0.6 mm posterior to the bregma, 2.0 mm below the skull surface, and 1 mm lateral to the bregma.

### 4.4 Treatments

For cromolyn sodium treatment, HFD-fed mice implanted with a guide cannula targeting the lateral ventricle were administered cromolyn sodium (MCE, HY-B0320A) intracerebroventricularly (i.c.v.) daily at a dose of 10 µg, immediately before the light was turned off. For administration of the SHU9119 (MCE, HY-P0227), lateral ventricle-cannulated HFD mice were briefly fasted, and received i.c.v. administration of SHU9119 (1µg,1 µL) or 0.9% NaCl as a control. After 1h and before lights were turned off, cromolyn sodium (10 µg, 1 µL) or NaCl was injected. Food intake and body weight were measured. To eliminate microglia, mice were received PLX5622(MCE, HY-114153) in drink water at 90 mg/kg once a day for 7 consecutive days.

### 4.5 Immunofluorescence

For immunofluorescence, mice were anesthetized with an intraperitoneal injection of avertin (300 mg/kg, Sigma). The brain was perfused and fixed with 4% paraformaldehyde (PFA). Brain tissue was dissected and post-fixed in 4% PFA for 4 hours, then dehydrated in 20% and 30% sucrose solutions at 4°C overnight. Using a cryostat (CM1950, Leica), 25 μm-thick sections were prepared. The sections were blocked in 5% serum diluted in 0.3% Triton X-100/PBS and incubated overnight at 4°C with primary antibodies, including anti-POMC (1:100,Phoenix Pharmaceuticals), anti-c-Fos (1:1000, Synaptic Systems), anti-Iba1 (1:1000, Abacm), anti-p-STAT3 (1:200, CST), and anti-α-MSH (1:100, Millipore). Sections were then incubated with fluorescent secondary antibodies at room temperature for 1 hour. Images were captured with an LSM980 confocal microscope (Carl Zeiss, Jena, Germany) and analyzed using ImageJ (Ver.1.8, NIH, Bethesda, MD). Cells in the representative ARC area on one side of each mouse’s brain section were manually counted.

### 4.6 Measurement of metabolic indicators

The body composition of mice was measured using DEXA (Analyzer, Seoul, South Korea). A metabolic monitoring system (Columbus Instruments, St. Paul, USA) was used to assess animal activity, food intake, oxygen consumption, carbon dioxide production, and energy expenditure, which were normalized to body weight. After cromolyn treatment, a glucose tolerance test (GTT) was conducted. Mice were fasted for 12–16 hours, followed by an intraperitoneal injection of glucose (1.5 g/kg body weight, dissolved in saline; Sigma, G8270, USA). Blood glucose levels were measured at 0, 15, 30, 60, 90, and 120 minutes using a glucometer (Roche, Basel, Switzerland).

### 4.7 Histological analysis

Samples of sWAT, eWAT, and liver tissue were fixed in 4% paraformaldehyde (PFA; Sangon Biotech, A500684, China). Tissue sections were cut at a thickness of 5 μm and stained with hematoxylin and eosin (H&E) (Servicebio, G1003, China). All quantitative analyses were conducted using Image-Pro Plus (Ver. 6, Media Cybernetics, Rockville, MD, USA).

### 4.8 Immunohistochemistry (IHC) analysis

For immunohistochemistry, mice were anesthetized with an intraperitoneal injection of avertin (300 mg/kg, Sigma) and perfused transcardially with 4% paraformaldehyde (PFA). Brain tissues were isolated and post-fixed in 4% PFA for 4 hours, followed by dehydration in 20% and 30% sucrose solutions at 4°C overnight. The tissues were sectioned at 25 μm thickness using a cryostat (CM1950, Leica) and stored at -80°C until use. The sections were processed for mast cell tryptase immunohistochemistry as follows: After incubation in 0.01 M PBS containing 10% bovine serum albumin and 0.3% Triton X-100 for 1 hour, tissue sections (30 μm) were incubated overnight at 4°C with the primary antibody for mast cell tryptase (1:100, Abcam, USA), followed by counterstaining with hematoxylin.

### 4.9 Statistical analysis

All data are presented as mean ± SEM. Analyses were performed using Prism 9 (GraphPad Software, San Diego, CA, USA). Comparisons between two groups were conducted using two-tailed Student’s t-test. For comparisons among more than two groups, one-way or two-way ANOVA was used, followed by Bonferroni’s *post hoc* test. A p-value of < 0.05 was considered statistically significant.

## Data Availability

The original contributions presented in the study are included in the article/supplementary material. Further inquiries can be directed to the corresponding authors.
